# Impaired eye movements in Parkinson's disease and their relationship to top–down and bottom–up neural processing

**DOI:** 10.1002/brb3.3404

**Published:** 2024-02-04

**Authors:** Nobuhiro Takahashi, Mimpei Kawamura, Yasutaka Kobayashi, Masahito Hitosugi

**Affiliations:** ^1^ Department of Rehabilitation, Faculty of Health Science Fukui Health Science University Fukui Japan; ^2^ Fukui Higher Brain Dysfunction Support Center Fukui Japan; ^3^ Department of Medical Welfare, Faculty of Health Sciences Kyoto Koka Women's University Kyoto Japan; ^4^ Department of Legal Medicine Shiga University of Medical Science Otsu Japan

**Keywords:** bottom–up and top–down processing, eye movement, Parkinson's disease

## Abstract

**Introduction:**

Eye movement disorder in patients with Parkinson's disease (PD) is less likely to appear when observing complex images compared with simple images. However, in case of auditory stimuli, meaningful stimuli can promote PD movement. Thus, we measured visual search movements with an eye tracker to investigate whether visual search changes in PD depending on the meaningfulness of visual stimuli; additionally, we measured event‐related potentials (ERPs) to neurophysiologically examine visual information processing in PD.

**Methods:**

Data from 11 patients with cognitively unimpaired PD and 10 neurologically healthy individuals (controls) were used in this study. We simultaneously measured eye tracking and ERPs during the observation of three types of images: operation, noun, and meaningless figures. We compared intergroup differences in visual search parameters, such as saccade count, fixation time, and saccade amplitude, as well as in ERPs vertex‐to‐vertex amplitudes and latency among P1, N1, P2, and N2 at the posterior regions. These ERPs reflect different stages of visual information processing and provide insights into the underlying neural mechanisms of visual search in PD.

**Results:**

All visual search parameters were consistently smaller in the PD group than in the control group, regardless of visual stimulus. The saccade count in the PD group was significantly lower than in the control group for operation and meaningless figures but not for noun figures. Further, P1 and N1 amplitudes—bottom–up processing—were smaller in PD group when viewing operation figures and N2 amplitudes—top–down processing—were larger in PD group when viewing operation and meaningless figures.

**Conclusions:**

PD's visual search movements may change depending on the meaningfulness of visual stimuli. Further, the abnormal visual search movements in PD may be due to insufficient bottom–up processing and excessive top–down processing.

**Plain Language Summary:**

Parkinson's disease (PD) is a neurodegenerative disorder that can cause eye movement disorders. People with PD may have difficulty searching for and processing visual information. In this study, we investigated how meaningfulness affects visual search movements and visual information processing in people with PD. We measured eye movements and brain activity in response to different types of visual stimuli, including meaningful and meaningless images, and compared them to a group of healthy control participants. Our results showed that PD patients had fewer eye movements and different brain activity patterns compared to healthy controls, regardless of the meaningfulness of the visual stimuli. However, we also found that the meaningfulness of visual stimuli had an effect on visual search movements in PD patients. This suggests that meaningfulness can impact the way people with PD process visual information. Understanding these differences could help in the development of new therapies to improve visual processing in people with PD.

## Graphical abstract

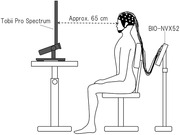

## 1 INTRODUCTION

When perceiving the outside world, humans repeatedly engage in active, rapid eye movements called saccades, followed by short fixation periods. Saccades intend to capture the object of interest onto the retinal fovea to obtain visual signals, whereas the short fixations serve to analyze and recognize the signals on the retina (Schall & Thompson, 1999). Thus, visual perception is achieved through a repeated process of saccades and fixation‐based visual signal processing.

Parkinson's disease (PD) is a progressive neurodegenerative disorder characterized by motor symptoms, such as bradykinesia, resting tremor, and rigidity (Postuma et al., 2015). However, PD also exhibits impairments in eye movements (Hikosaka et al., 2000; Yugeta et al., 2010). Additionally, among other nonmotor symptoms of PD (Borek et al., 2006; Thanvi et al., 2003), there is an early decline in visual processing ability (Armstrong, 2015, 2017). Based on clinical observations, this decline in visual processing ability in PD has been considered to result from eye movement dysfunction (Crawford et al., 1989; Shibasaki et al., 1979). However, recent neuropsychological evaluations have revealed that PD exhibits a decline in visual information processing functions, such as visual working memory (Hodgson et al., 1999; Lee et al., 2010; Zhao et al., 2018) and visual spatial perception (Leek et al., 2014; Montse et al., 2001). Accordingly, the decline in visual cognition in PD may not only be caused by eye movement dysfunction but also by higher‐level visual information processing impairments. Thus, to identify the underlying functional impairments, simultaneous measurements of eye movements and brain neural activity are necessary. This result could contribute to the development of diagnostic and therapeutic approaches for PD in the future. Furthermore, similar approaches could be applied to other diseases related to visual cognition, which may help to elucidate the underlying mechanisms of those diseases.

On the other hand, the eye movement abnormalities observed in PD are less likely to occur when viewing complex figures compared to simple ones (Matsumoto et al., 2011). However, in this study, meaningful and meaningless figures were mixed in the visual stimuli, so the involvement of meaning cannot be ruled out. Sacks reported that familiar music can induce PD movements (Sacks, 1973). Furthermore, the auditory stimuli that induce in PD are supposedly meaningful (Distler et al., 2016). A study using cataleptic rats treated with haloperidol showed that movement was facilitated by using auditory stimuli that were part of their communication repertoire, that is, meaningful stimuli for the rats (Tonelli et al., 2017). Based on these findings, the meaningfulness of visual stimuli may also influence visual exploration in PD.

Event‐related potentials (ERPs) related to linguistic semantic processing have traditionally been associated with the N400 component (Kutas & Hillyard, 1980). However, ERPs, such as P1, N1, P2, and N2, which are observed at earlier latencies than N400, have been involved in nonlinguistic semantic processing, such as category discrimination with visual information (Proverbio et al., 2007; Zani et al., 2015). Further, studies on ERPs examining visual information processing in PD patients and healthy individuals have reported differences in amplitude and latency between groups in the early ERP components, including P1, N1, P2, and N2 in the posterior region of the brain (Antal et al., 2003; Li et al., 2003; Wang et al., 2001).

Normal visual cognition requires a balance between top–down and bottom–up processing. Top–down processing attempts to fit sensory input into one's own abstract concepts, whereas bottom–up processing sends out actual sensory input (Mumford, 1992). ERP studies on visual information processing have been suggested that P1 and N1 are influenced by bottom–up processing and reflect external attention selection mechanisms, whereas N2 is influenced by top–down processing and is an internally generated component related to stimulus evaluation and classification (Shedden & Nordgaard, 2001).

In this study, we used an eye tracker to measure visual search movements in individuals with PD to investigate whether a visual search is influenced by the meaningfulness of the visual target. We simultaneously measured the early ERP components, which have been associated with nonlinguistic visual information processing, namely, bottom–up and top–down processing, to neurologically examine visual information processing in individuals with PD. There have been reports that individuals with PD are more likely to have difficulty with verb processing in language tasks (Cardona et al., 2013; Garc´ıa & Ibáñez, 2014). Therefore, given the possibility of differences in the processing of noun and operation figures during visual information processing, the meaningful figures in this study were analyzed separately as two types: noun and operation figures.

## 2 MATERIALS AND METHODS

### 2.1 Subjects

The PD group included consecutive patients with PD who received rehabilitation at the Department of Rehabilitation Medicine at Fukui General Hospital between March and August 2021. The inclusion criteria for the PD group were as follows: (i) diagnosis by a neurologist based on the Movement Disorder Society Clinical Diagnostic Criteria for PD (Postuma et al., 2015), (ii) Hoehn and Yahr stage between 1 and 3, and (iii) ability to maintain a stable seated position on a chair or wheelchair with a backrest during the experiment. The exclusion criteria were as follows: (i) obvious abnormalities in visual acuity or visual field, (ii) undergoing deep brain stimulation therapy, (iii) organic brain lesions, and (iv) dementia. Participants with dementia were excluded based on the Revised Hasegawa's Dementia Scale (HDS‐R) (Imai & Hasegawa, 1994), a simple intelligence test primarily used for screening dementia. HDS‐R is commonly used in Japan along with the mini–mental state examination and proven useful in detecting dementia (Kim et al., 2005). The total HDS‐R score was 30 points; scores ≤20 indicate a higher likelihood of dementia. In the end, 11 patients with PD (8 males and 3 females) were recruited for the study (Table 1). All patients with PD were taking antiparkinsonian drugs; the experiment was conducted in the ON state for each patient.

**TABLE 1:** Characteristics of Parkinson's disease (PD) patients and control subjects.

		PD patient *n* = 11	Control *n* = 10	*p*
Sex	Male/Female	8/3	6/4	.536
Age	Years	70.7 (8.6)	69.5 (3.9)	.685
HDS‐R score	Points	28 (27.0–29.0)	29 (28.3–29.8)	.217

*Note*: Age: average (standard deviation, SD), hierarchic dementia scale‐revised (HDS‐R) score: median (interquartile range, IQR). Sex: Chi‐squared test, age: Student's *t*‐test. HDS‐R score: Mann–Whitney *U* test.

The control group consisted of 10 orthopedic patients (6 males and 4 females) who underwent shoulder surgery and were hospitalized at Fukui General Hospital from November to December 2021. These orthopedic patients had no history of psychiatric or neurological disorders and were considered neurologically healthy. Informed consent was obtained from all participants for their participation in the study was conducted in accordance with the ethical standards of the Helsinki Declaration and approved by the Ethics Committee of the Nittazuka Medical Welfare Center (Nittazuka Ethics 2020‐18).

### 2.2 Simultaneous measurement of eye gaze and ERPs

**FIGURE 1:**
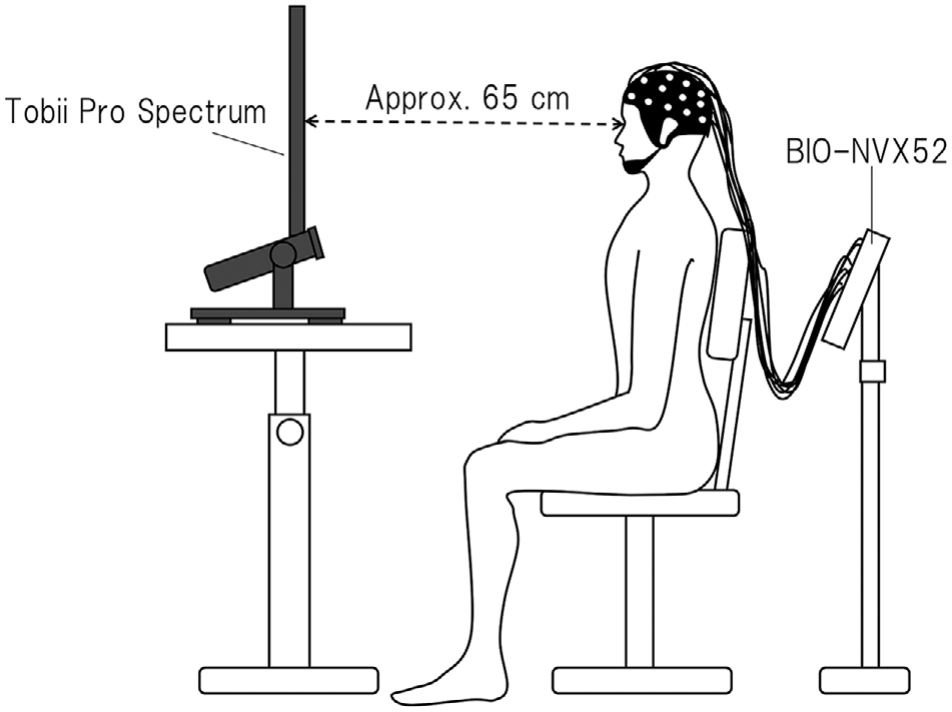
Experimental environment of the study.

**FIGURE 2:**
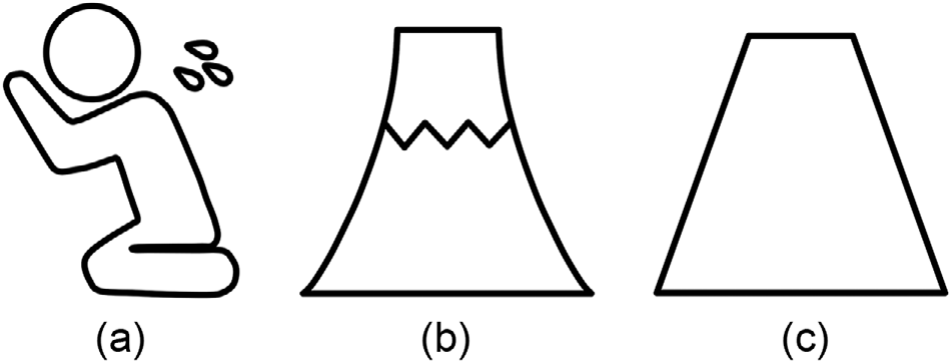
Examples of visual stimuli: (A) operation figures; (B) noun figures; (C) meaningless figures.

We simultaneously measured eye movements and ERPs during viewing of various images using the following procedure:

The participants were seated at a distance of approximately 65 cm from a monitor for visual stimulation. The monitor was placed on an adjustable table to match the participant's eye level (Figure 1). We used the screen‐based eye tracker Tobii Pro Spectrum (Tobii AB) and the Tobii Pro Lab software (Tobii AB) for biological measurement and analysis and obtained binocular eye gaze data at a sampling rate of 600 Hz. Due to its special stereo image processing, the eye tracker used in this study maintained the accuracy of eye position and gaze estimation even when the position of the head changed. Therefore, we did not fix the participants' heads with a chinrest or other device. Before measuring eye gaze data, we performed a nine‐point calibration to match the participant's gaze position with the monitor coordinates. The participant's performance was displayed in real‐time on a second monitor, which was continuously monitored. We classified eye gaze data with angular velocities <30° as fixation and those ≥30° as saccades. The experimental design for visual stimulation was created using Tobii Pro Lab (Tobii AB) and presented on a 23.8‐in. monitor (1920 × 1080 pixels). A central fixation point was shown for 1000 ms before each image was presented for 4000 ms in a random order for a total of 90 images across three conditions (30 images per condition): operation, noun, and meaningless figures (Figure 2). The participants were instructed to look at any part of the images.

ERPs were measured using the DC digital electroencephalogram system BIO‐NVX52 (Medical Computer Systems) and the recording software NeoRec (Medical Computer Systems), according to the international 10–20 system, with recordings in 19 channels. The average potential of A1 and A2 was used as the reference electrode. The channels were amplified in the frequency range of direct current to 40 Hz. The sampling rate was set to 250 Hz, and the impedance was maintained <5 kΩ. Artifact removal was performed using the eye movement artifact control method (Pfeifer et al., 1995). The timing of visual stimulation was used as a trigger for ERPs. The trigger signal was captured by a light sensor (East Medic Corporation) attached to the monitor for visual stimulation and precisely synchronized with the eye tracker.

### 2.3 Analysis

We conducted group comparisons on participant characteristics using the Chi‐squared test for sex, the Student's *t*‐test for age, and Mann–Whitney *U* test for HDS‐R score. As parameters for visual exploration, we set the average number of saccades (number of saccades during a 4000 ms visual stimulus presentation; *n*), average fixation duration (average time from the end of one saccade to the start of the next; ms), and average saccade amplitude (°). We performed a two‐way analysis of variance (ANOVA) (3 × 2) using image type (operation, noun, and meaningless figures) and disease (PD, control) for each parameter. If the main effect of the disease was significant, we conducted post hoc group comparisons using a Student's *t*‐test for each image type.

For ERPs, we analyzed the ch‐O1 and O2 regions in the occipital lobe and calculated the mean amplitude and latency between the peaks for the initial ERPs components, P1 (70–140 ms), N1 (160–190 ms), P2 (200–250 ms), and N2 (260–300 ms). We performed a two‐way ANOVA (3 × 2) with image type and disease for the amplitude and latency between the peaks of each ERP component. If the main effect of the disease was significant, we conducted post hoc group comparisons using a Student's *t*‐test for each image type.

We used Statcel‐the Useful Addin Forms on Excel—4th ed. (OMS Publishing Co) for all statistical analyses. The significance level for all statistical analyzes was set at 5% but we corrected the significance level using the Bonferroni method (*p* < .016) for post hoc Student's *t*‐test to account for multiple comparisons.

## 3 RESULTS

### 3.1 Subjects characteristics (Table 1)

There was no significant difference in the proportion of male and female participants among groups. The mean age (standard deviation) of the PD group was 70.7 (8.6) years and that of the control group was 69.5 (3.9) years, without significant differences between groups. The median (interquartile range) HDS‐R score was 28 (27.0–29.0) points did not differed significantly from that of the control group (29 [28.3–29.8] points).

### 3.2 Eye tracking (Figure 3, Table 2)

Regardless of image type, the three parameters of the visual search consistently showed smaller values in the PD group than in the control group.

There was no interaction between the image type and disease for the saccade number. Although there was no main effect of image type, a significant main effect of disease (*p* < .001) was observed. Post hoc comparisons showed a significantly lower number of saccades in the PD group that in the control group for operation figures (*p* < .016) and meaningless figures (*p* < .016), but not for noun figures (*p* = .033).

There was no interaction between image type and disease, no main effect of image type, and no main effect of disease for fixation time. Similarly, there was no interaction between image type and disease, no main effect of image type, and no main effect of disease on saccade amplitude.

**FIGURE 3:**
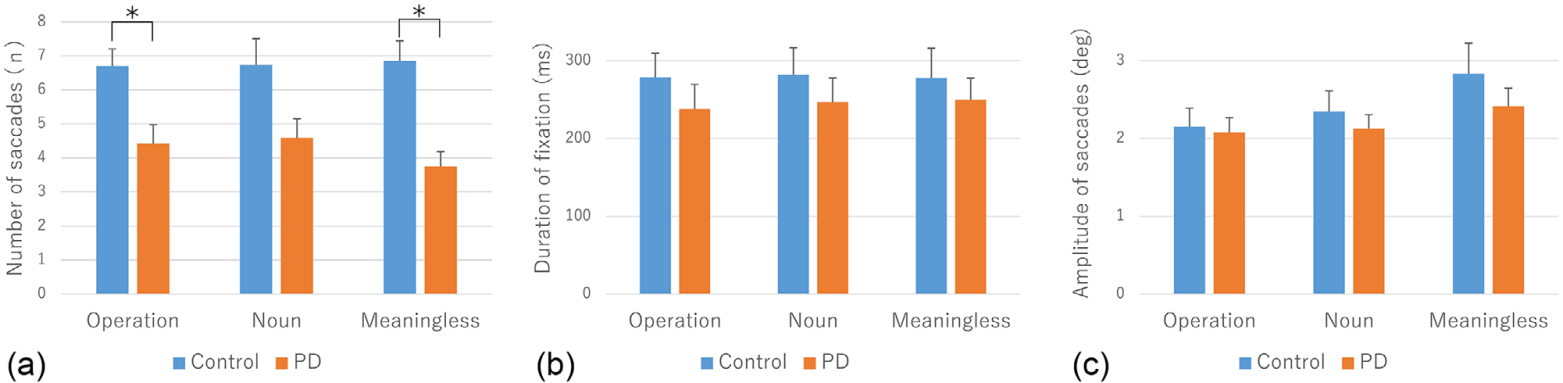
Visual scanning parameters in control subjects and patients with Parkinson's disease (PD): (A) number of saccades; (B) duration of fixation; (C) amplitude of saccades. The bars show the values of mean and standard error. *p < .016 (correction for multiple comparisons by Bonferroni's method).

**TABLE 2:** Results of two‐way analysis of variance (ANOVA) for eye tracking and event‐related potentials (ERPs).

				Main effects		Interactions
				Picture type (Operation, noun, meaningless)	Disease (PD, Control)	Picture type × disease
**Eye tracking**		Number of saccades		*F*(2, 61) = .262, *p* = .770	*F*(1, 61) = 28.422, *p* < .001	*F*(2, 61) = .406, *p* = .667
		Duration of fixation		*F*(2, 62) = .024, *p* = .975	*F*(1, 62) = 1.695, *p* = .198	*F*(2, 62) = .019, *p* = .981
		Amplitude of saccades		*F*(2, 61) = 2.095, *p* = .132	*F*(1, 61) = 1.295, *p* = .278	*F*(2, 61) = .234, *p* = .791
**ERPs**	**Amplitude**		P1	*F*(2, 121) = 7.732, *p* < .001	*F*(1, 121) = 6.221, *p* < .05	*F*(2, 121) = 2.427, *p* = .092
			N1	*F*(2, 120) = 1.602, *p* = .205	*F*(1, 120) = 5.472, *p* < .05	*F*(2, 120) = .020, *p* = .980
			P2	*F*(2, 120) = .011, *p* = .989	*F*(1, 120) = 7.923, *p* < .01	*F*(2, 120) = .010, *p* = .989
			N2	*F*(2, 117) = .456, *p* = .634	*F*(1, 117) = 8.275, *p* < .01	*F*(2, 117) = 1.925, *p* = .151
	**Latency**		P1	*F*(2, 102) = 3.171, *p* < .05	*F*(1, 102) = 4.846, *p* < .05	*F*(2, 102) = 2.251, *p* = .111
			N1	*F*(2, 120) = .532, *p* = .588	*F*(1, 120) = 2.543, *p* = .113	*F*(2, 120) = .286, *p* = .751
			P2	*F*(2, 120) = .269, *p* = .764	*F*(1, 120) = 2.124, *p* = .147	*F*(2, 120) = 3.449, *p* < .05
			N2	*F*(2, 116) = 2.898, *p* = .059	*F*(1, 116) = .991, *p* = .321	*F*(2, 116) = 2.363, *p* = .987

### 3.3 ERPs

#### 3.3.1 ERP amplitude (Figure 4, Table 2)

There was no significant interaction between image type and disease in P1 amplitude. A significant main effect was found for image type (*p* < .001) and disease (*p* < .05). Post hoc tests showed a significantly smaller P1 amplitude in the PD group than in the control group for operation figures (*p* < .016), but no significant differences were observed for noun figures (*p* = .087) and meaningless figures (*p* = .812).

There was no significant interaction between image type and disease, or a main effect of image type in N1 amplitude, but a significant main effect was found for disease (*p* < .05). Post hoc tests showed no significant differences between groups for operation figures (*p* = .027), noun figures (*p* = .197), and meaningless figures (*p* = .067), although a small *p* value was found for operation figures, indicating that the N1 amplitude in the PD group tended to be smaller than that in the control group.

There was no significant interaction between image type and disease or the main effect of image type in P2 amplitude, but a significant main effect was found for disease (*p* < .01). Post hoc tests showed no significant differences between groups for all types of figures (operation figures: *p* = .107, noun figures: *p* = .087, and meaningless figures: *p* = .148).

Similarly, there was no significant interaction between image type and disease, or the main effect of image type in N2 amplitude, but a significant main effect was found for disease (*p* < .01). Post hoc tests showed no significant differences between groups for noun figures (*p* = .808) and meaningless figures (*p* = .027), but the N2 amplitude in the PD group was significantly larger than that in the control group for operation figures (*p* < .016). Thus, a small *p* value for meaningless figures indicates that the N2 amplitude in the PD group tended to be larger than that in the control group.

**FIGURE 4:**
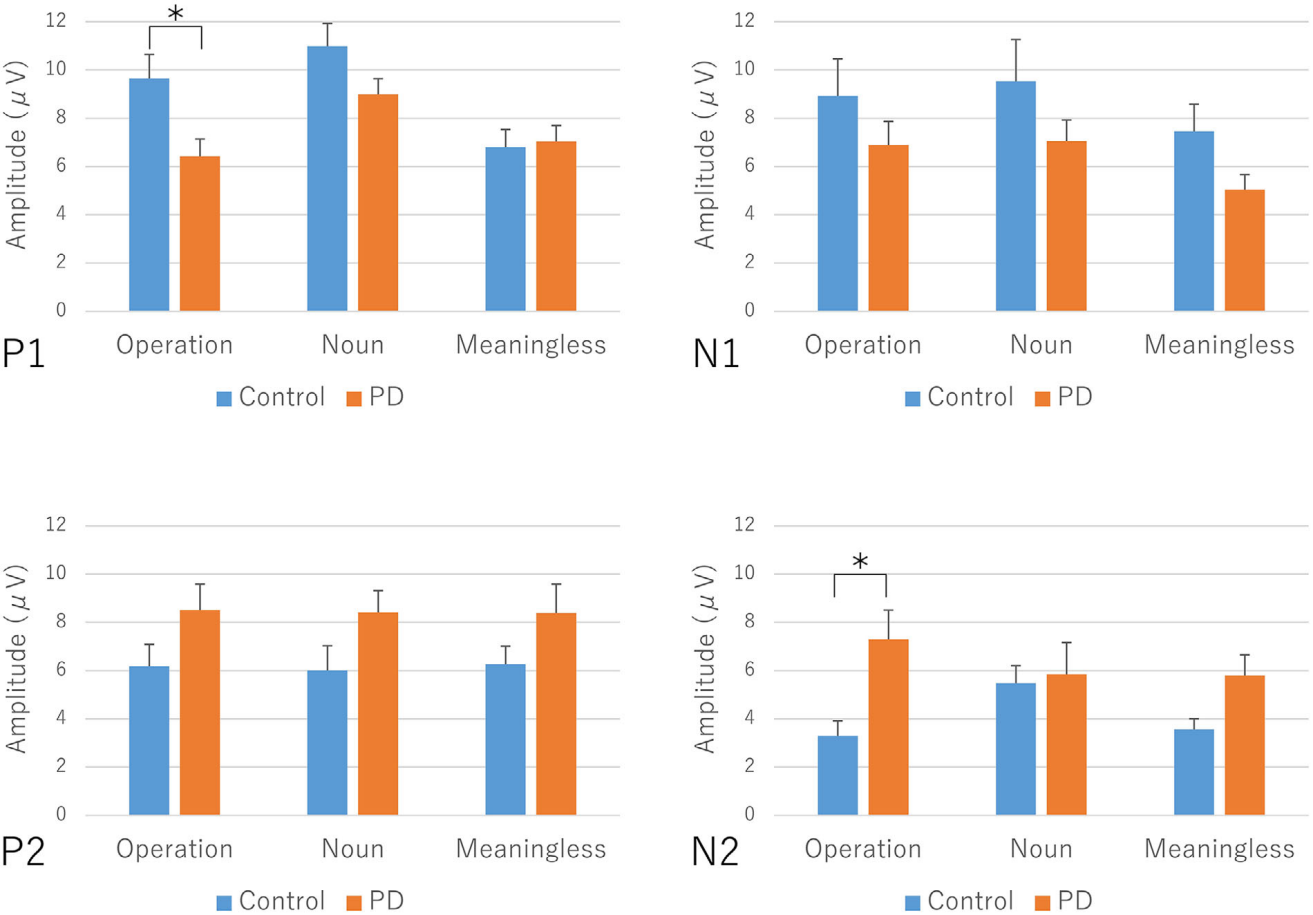
Event‐related potentials (ERPs) in control subjects and patients with Parkinson's disease (PD). The bars show the values of mean and standard error. *p < .016 (correction for multiple comparisons by Bonferroni's method).

#### 3.3.2 ERP latency (Table 2)

There was no interaction between image type and disease for P1 latency but a main effect was found for image type (*p* < .05) and disease (*p* < .05). Post hoc tests revealed no significant between‐group differences for any image type, including operation figures (*p* = .614), noun figures (*p* = .074), and meaningless figures (*p* = .060).

For N1 latency, no interaction between image type and disease or main effects was detected. Similarly, no interaction or main effects were found for N2 latency for image type and disease.

An interaction between image type and disease was found for P2 latency (*p* < .05), but no main effects were observed for image type or disease.

## 4 DISCUSSION

### 4.1 Eye movement disorders in PD

The results of the eye‐tracking analysis showed that, compared with controls, PD patients consistently demonstrated lower values for all three visual search parameters, regardless of image type. Several studies have evaluated eye movements during visual search tasks in patients with central nervous system disorders. For example, in PD, the fixation area during visual search tasks for shape recognition is narrower (Matsumoto et al., 2011) but wider in hereditary ataxia (Matsuda et al., 2015). These results suggest that impairments of specific motor function affect visual search. The findings from current study support previous research showing that bradykinesia in PD affects both limb and eye movement (Matsumoto et al., 2011; Shibasaki et al., 1979). However, a narrow range of visual search has been also observed in diseases such as schizophrenia (Kojima et al., 1990; Suzuki et al., 2009; Takahashi et al., 2008) and Alzheimer's dementia (Nakashima et al., 2010), which do not involve motor restrictions. Therefore, cognitive decline may also affect eye movements. PD is a central nervous system disorder that causes both motor and cognitive impairment (Barone et al., 2009). It has been reported that PD has visual system problems from the early stages of cognitive function (Armstrong, 2017). Although there was no difference in HDS‐R scores between patients with PD and controls in this study, the decrease in visual search ability observed in PD may reflect not only pure motor impairment but also limited visual cognitive function.

### 4.2 PD eye movements and ERPs for different types of visual stimuli

Our gaze analysis results identified significant differences in the saccade count between the PD and control groups depending on the type of visual stimuli, whereas others showed no significant differences. It is believed that the visual search ability of PD changes with the differences in visual stimuli, approaching that of healthy individuals, and cannot be explained solely by pure motor impairment. Matsumoto et al. (2011) observed a reduced impairment of visual search in PD, similar to that of healthy individuals with more complex visual stimuli. Based on our results, the addition of meaning to visual stimuli may also change PD's visual search behavior.

Among the two types of meaningful stimuli (operation and noun figures), there were no significant group differences in the three visual search parameters or the amplitude and latency of each ERP while looking at noun figures, indicating that both PD patients and controls had similar visual search behavior and neural processing when looking at pictures of common nouns. There was a significantly lower number of saccades looking at operation figures in PD patients than in controls. The P1 amplitude was significantly smaller and the N2 amplitude significantly larger in PD while looking at operation figures. Rodríguez‐Ferreiro et al. (2009) showed that while object and action naming scores were similar in patients with Alzheimer's disease (AD), patients with PD had a significant impairment in action naming compared with object naming. Furthermore, Abrevaya et al. (2017) compared the brain activity during verb and noun processing between PD and healthy individuals using functional magnetic resonance imaging. No significant difference in brain activity was observed between groups during noun processing; however, during verb processing, healthy individuals used the motor network in the front of the brain to process verbs, whereas PD used the nonmotor network at the back of the brain.

Both noun and operation figures are meaningful visual stimuli; the differences in visual search movements and ERPs observed may be related to the previously reported differences in PD's verb and noun processing abilities (Abrevaya et al., 2017; Rodríguez‐Ferreiro et al., 2009). In this study, there was no input of linguistic stimuli and no demand for linguistic output. However, the differences observed in this study suggest that PD may be less impaired in processing noun figures than in processing operation figures not only at the linguistic level but also at the visual cognitive level.

### 4.3 Relationship between visual cognitive impairment in PD and bottom–up and top–down processing

The visual hallucinations frequently observed in PD have been related to dysfunction in both bottom–up perception from external stimuli and top–down prediction from internally generated visual imagery (Armstrong, 2011; Collerton et al., 2005; Fenelon, 2008; Llebaria et al., 2010; Onofrj et al., 2007; Sanchez‐Castaneda et al., 2010; Shine et al., 2011). Although P1 and N1 are influenced by bottom–up processing, N2 is suggested to be influenced by top–down processing (Shedden & Nordgaard, 2001). In this study, the N2 amplitude in the PD group was significantly larger than that in the control group for operation figures, but both groups had similar amplitudes for noun figures. Although no significant between‐group differences were observed for meaningless figures, the N2 amplitude tended to be larger in the PD group. The larger N2 amplitude in PD may reflect excessive top–down processing. Although meaningless figures require less top–down processing, the N2 amplitude was still large in PD. This may be due to pareidolia, a visual phenomenon where clear and specific illusions are perceived from unclear or meaningless visual objects (Blom, 2010), and which is highly prevalent in PD (Uchiyama et al., 2015). In PD, even meaningless stimuli can trigger excessive top–down processing, leading to visual hallucination. For operation figures, the N2 amplitude in PD was significantly larger and the P1 amplitude significantly smaller than in the control group. Although no significant between‐group differences were observed for N1 amplitude, it tended to be smaller in the PD group than in the control group. Dalla et al.’s ERPs study on the hierarchical processing of verbs in healthy individuals suggested that the sensory‐motor circuit from the frontal to parietal regions is involved in bottom–up processing, whereas the posterior temporal and parietal regions contribute to top–down processing (Volta et al., 2018). PD makes it difficult to process verbs (Rodríguez‐Ferreiro et al., 2009), and it has been previously mentioned that processing verbs using the motor network in the frontal brain is difficult and that nonmotor networks are used for processing (Abrevaya et al., 2017). The smaller P1 and N1 amplitudes in PD observed in this study may indicate restricted use of the motor network in the frontal brain for verb processing and insufficient bottom–up processing. In contrast, the larger N2 amplitude may indicate excessive top–down processing using nonmotor networks in the parietal and posterior temporal regions.

The preserved ability of patients with PD to overcome their compromised motor control (such as slowness of movement and decreased movement range) under specific environmental circumstances is often referred to as paradoxical kinesia (Asmus et al., 2008) or akinesia paradoxical (Heilman & Valenstein, 2011). The neural mechanism of paradoxical kinesia is postulated to occur as a result of movements induced by external stimuli bypass pathways in the degenerated basal ganglia (Martin, 1967). We examined the improvement of eye movements in patients with PD using noun pictures in terms of bottom–up and top–down processing. We propose this as an improvement in motor function by a different mechanism than the conventional paradoxical kinesia expression hypothesis.

Several eye‐tracking studies have examined the visual cognitive function of patients with neurodegenerative diseases from the perspective of top–down and bottom–up processing. Rösler et al. (2000) reported that patients with AD were unable to perform top–down visual search strategies and had an increased number of fixations. Similarly, in primary progressive aphasia (PPA), there is an increase in the number of saccades in tasks involving word‐object matching (Seckin et al., 2016) and picture naming tasks (Ungrady et al., 2019). These results evidence that bottom–up processing becomes dominant due to deficient top–down processing. In this study, the number of saccades made by patients with PD while looking at noun figures was similar to that of controls but significantly decreased when observing operation and meaningless figures. This lower number of saccades is thought to reflect excessive top–down processing, consistent with the ERP results. Our study, which focused on patients with PD, revealed a mechanism different from those observed in previous studies on AD and PPA. This novel finding suggests that the visual cognitive function of patients with PD is distinct from that of patients with AD or PPA.

### 4.4 Limitations

First, the number of participants was relatively small. However, we believe that our results are valid as we conducted appropriate statistical processing. Second, the results may differ depending on the time of day or day of the week the experiment was conducted. PD is characterized by symptom fluctuations (Videnovic & Willis, 2016), and it is uncertain whether participants were in their best condition when they participated in the study. However, we adjusted the time of day as much as possible to allow PD patients to participate in the study in the ON state. Third, we were unable to perform a detailed assessment of nonmotor symptoms of PD, including motor symptoms and visual hallucinations. If we had been able to obtain these data, we might have been able to better clarify the relationship among visual search parameters, ERP results, and clinical symptoms of PD. Fourth, although relatively simple images were used for all types of figures, there is a limitation in fully controlling the image complexity. The noun figures used in this study were slightly simpler in shape than the operation figures but the saccade number of PD patients was lower than that of controls for operation figures, and equal for noun figures. Fifth, this study differs from previous eye tracking studies with PD in that participants were not given explicit instructions other than to look at a picture. However, it is important to note that method of freely looking at pictures in this study revealed that eye movements and ERPs during unconscious visual cognitive processing differed between patients with PD and healthy participants. Finally, can we conclude that the operation, the noun, and the meaningless figure used in this experiment are representative of each of them? For example, there are differences in brain regions associated with the processing of nouns depending on semantic category, such as names, animals, and tools (Damasio et al., 1996, 2004). As such differences may affect PD's visual cognition, further detailed studies are needed in the future.

## 5 CONCLUSIONS

Although the visual search movements of PD patients were not significantly different from those of controls when viewing noun figures, they had significantly fewer saccades when viewing operation or meaningless figures. According to our ERP results, PD seems to affect visual search movements by excessive top–down processing when viewing operation or meaningless figures.

## AUTHOR CONTRIBUTIONS

Nobuhiro Takahashi, Mimpei Kawamura, and Yasutaka Kobayashi designed the study. Nobuhiro Takahashi and Mimpei Kawamura performed the research. Yasutaka Kobayashi and Masahito Hitosugi aided and advised on eye tracking and ERP measurement and analysis. Nobuhiro Takahashi, Mimpei Kawamura, and Masahito Hitosugi analyzed the data. Nobuhiro Takahashi and Mimpei Kawamura wrote the manuscript. All authors contributed to editorial changes in the manuscript. All authors read and approved the final manuscript.

## CONFLICT OF INTEREST STATEMENT

The authors declare no conflicts of interest.

## FUNDING INFORMATION

This research received no external funding.

### PEER REVIEW

The peer review history for this article is available at https://publons.com/publon/10.1002/brb3.3404.

## Data Availability

The data that support the findings of this study are available from the corresponding author upon reasonable request.
